# Free‐ranging livestock affected the spatiotemporal behavior of the endangered snow leopard (*Panthera uncia*)

**DOI:** 10.1002/ece3.9992

**Published:** 2023-04-19

**Authors:** Jiaxin Li, Xiaogang Shi, Xingcheng He, Dongrui Li, Qiang Hu, Yanni Zhang, Jianghong Ran

**Affiliations:** ^1^ Key Laboratory of Bioresources and Eco‐Environment of Ministry of Education, College of Life Sciences Sichuan University Chengdu Sichuan China; ^2^ Wolong National Nature Reserve Administration Bureau of Wolong National Nature Reserve Wolong Town, Wenchuan County Aba China

**Keywords:** blue sheep (*Pseudois nayaur*), grazing disturbance, interspecific interactions, snow leopard (*Panthera uncia*)

## Abstract

Long recognized as a threat to wildlife, particularly for large carnivores, livestock grazing in protected areas can potentially undermine conservation objectives. The interspecific interactions among livestock, snow leopards (*Panthera uncia*), and their wild prey in fragile Asian highland ecosystems have been a subject of debate. We strategically deployed 164 camera traps in the Wolong National Nature Reserve to systematically investigate the activities of snow leopards, their primary wild ungulate prey species, and free‐ranging livestock. We found that snow leopard habitat use was influenced by both wild prey and livestock. Blue sheep served as the main wild prey that spatially attracted snow leopards and coexisted with yaks while free‐ranging yaks significantly restricted snow leopard habitat use both temporally and spatially. This study challenges the conventional understanding that livestock indirectly impacts large carnivores by competing with and displacing wild prey. Our findings highlight that free‐ranging yaks within the alpine canyon terrain could directly limit snow leopard habitat use, suggesting a potential risk of grazing in reducing apex predator distribution and jeopardizing their populations. Consequently, managing their coexistence in shared habitats requires a more nuanced approach. Furthermore, our research underscores the importance of further research efforts aimed at enhancing our comprehension of the complex interplay within animal communities and ecosystems. This knowledge will contribute to the development of informed, evidence‐based conservation strategies and policies.

## Graphical abstract

We explored the interspecific relations between snow leopard, blue sheep, and free‐ranging livestock. It is shown that the populations of three species reached coexistence through temporal, spatial, or other adaptations of their activities, suggesting the potential risk of grazing in reducing snow leopard distribution.

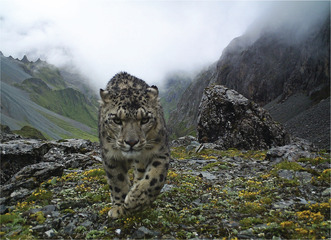

## 1 INTRODUCTION

Large carnivores, as apex predators, play pivotal ecological roles in maintaining the structure and function of ecosystems through the top‐down control of biomes (Brashares et al., 2010; Hoeks et al., 2020; Lindsey et al., 2007; Ripple et al., 2014). However, these apex predators are particularly susceptible to anthropogenic disturbances, experiencing significant population declines and distribution range contractions worldwide due to human activities such as land use, livestock grazing, poaching, retaliatory hunting, and fur trade (Ripple et al., 2014; Van Eeden et al., 2018; Wolf & Ripple, 2016). Empirically, livestock grazing, as a widespread disturbance globally, poses a pervasive threat to large carnivores (Pudyatmoko, 2017; Samia et al., 2015; Schieltz & Rubenstein, 2016; Schuette et al., 2013).

The relationship between grazing and large carnivores has become a central focus in large carnivore conservation research. Studies have shown that the impacts of livestock on large carnivore habitat use are complex and varied (Kafley et al., 2019; Pudyatmoko, 2017; Sharma et al., 2015; Soofi et al., 2018). On the one hand, livestock indirectly affects large carnivore activities by influencing their primary prey, wild ungulates (Kafley et al., 2019). Livestock can negatively impact wild ungulates in various ways, such as increasing the risk of disease transmission and the competition for food, habitat, and other resources (Hong et al., 2021; Schieltz & Rubenstein, 2016). However, some researchers argue that appropriate grazing can promote vegetation regeneration and improve the quality of edible plants, benefitting wild herbivore survival (Dave & Jhala, 2011; Khan, 1995). On the other hand, according to the alternative prey hypothesis, livestock may serve as alternative food resources for large carnivores when wild prey densities were low (Lack, 1954; Nordberg & Schwarzkopf, 2019), directly affecting large carnivores through interspecies predation (Thapa et al., 2021; Wilkinson et al., 2020). Livestock could sustain predator populations as vital food supplements (Kafley et al., 2019; Rovero et al., 2018; Shao et al., 2021; Sundararaj et al., 2012). Livestock constitutes essential food sources for vulnerable predators, especially those coexisting with multiple predators, allowing them to avoid competition for resources with dominant predators and achieve coexistence (Kafley et al., 2019). However, this positive effect can only be realized if livestock densities remain below a threshold (Sharma et al., 2015). Otherwise, overgrazing may lead to decreased wildlife occupancy and abundance (Feng et al., 2021; Pudyatmoko, 2017; Soofi et al., 2018; Wang et al., 2018). Simultaneously, economic losses from livestock predation by wild carnivores can increase hostility and even retaliatory killing by herders (Hussain et al., 2018; Johansson et al., 2015; Li & Lu, 2014), exacerbating the threat to large carnivores.

In summary, numerous factors influence large carnivore‐livestock interactions, including grazing practices (Maheshwari & Sathyakumar, 2020), grazing scale (Filla, Lama, Filla, et al., 2022, Filla, Lama, Ghale, et al., 2022; Sueur et al., 2014), wild prey availability (Lu & Bullock, 2021; Thapa et al., 2021; Wilkinson et al., 2020), and community cultural context (Shao et al., 2021). A lack of ecological data may hinder the effectiveness of carnivore conservation strategies development (Li, Bleisch, et al., 2020, Li, McShea, et al., 2020; Wang et al., 2014). Therefore, more regional studies are needed to elucidate the effects of grazing activities on large carnivores, facilitating the reconciliation of grazing and large carnivore conservation while providing scientific guidance for targeted conservation management.

The Snow leopard (*Panthera uncia*), an apex predator on the Qinghai‐Tibet Plateau (QTP), is classified as Vulnerable (VU) on the IUCN Red List of Threatened Species (McCarthy et al., 2017). As a local flagship species and umbrella species, snow leopards can contribute to alpine biodiversity conservation actions (Li, Bleisch, et al., 2020, Li, McShea, et al., 2020). Factors such as human disturbance and climate change (Cameron et al., 2016; Li et al., 2016) have led to declines in snow leopard populations and habitat ranges (McCarthy et al., 2017). Pastoralism constitutes the dominant anthropogenic disturbance in snow leopard habitats and has multifaceted effects on this species (Sharma et al., 2015). Snow leopards depend on wild ungulates such as blue sheep (*Pseudois nayaur*), ibex (*Capra sibirica*), and argali (*Ovis ammon*), among others, as their main prey (Johansson et al., 2015; Shao et al., 2021; Suryawanshi et al., 2017). The abundance and distribution of these ungulates decide the population dynamics and spatial patterns of snow leopards (Filla, Lama, Filla, et al., 2022, Filla, Lama, Ghale, et al., 2022; Sharief et al., 2022). Increasing grazing activities pressure wild ungulates (Karimov et al., 2018; Lu et al., 2021), potentially affecting the predation efficiency of large carnivores such as the snow leopard indirectly (Ghoshal et al., 2017; Yang et al., 2021). However, livestock has also been found to replace wild ungulates and fill the food needs of snow leopards occasionally (Rovero et al., 2018), sometimes even as the primary prey (Bocci et al., 2017). The effects of grazing on snow leopards remain controversial, necessitating further research to clarify the interactions among snow leopards, livestock, and wild ungulates.

In this study, we monitored the activities of free‐ranging livestock, snow leopards, and their wild prey (blue sheep) using camera traps in Wolong National Nature Reserve to investigate whether, and how grazing activities affect snow leopard habitat use. Our findings will contribute to understanding the adaptation strategies of snow leopards to grazing disturbances in different regions and serve as a scientific basis for reconciling grazing and snow leopard conservation. We hypothesize that snow leopard habitat use is primarily influenced by the distribution of their wild prey, while livestock may affect snow leopard movements by competitively excluding wild ungulates or serving as potential prey.

## 2 MATERIALS AND METHODS

### 2.1 Study area

The Wolong National Nature Reserve (WNNR) is located in Wenchuan County, Aba Tibetan and Qiang Autonomous Prefecture, Sichuan Province, in southwest China (102°2″–103°24″ E, 30°45″–31°25″ N). Established in 1963, the reserve lies at the eastern edge of the snow leopard distribution range and features an alpine‐gorge terrain, seated on the transition zone from the Chengdu Plain to the Qinghai‐Tibet Plateau. The terrain within the reserve gradually ascends from southeast to northwest, spanning an elevation of 5100 m (Cai & Huang, 1990). As the elevation increases, the vegetation in the reserve displays a distinct vertical distribution (Huang et al., 2007). The boundary between forest and scrub meadow lies at approximately 3800 m above sea level, while scree dominates the land cover type above 4400 m (Song et al., 2006).

**FIGURE 1:**
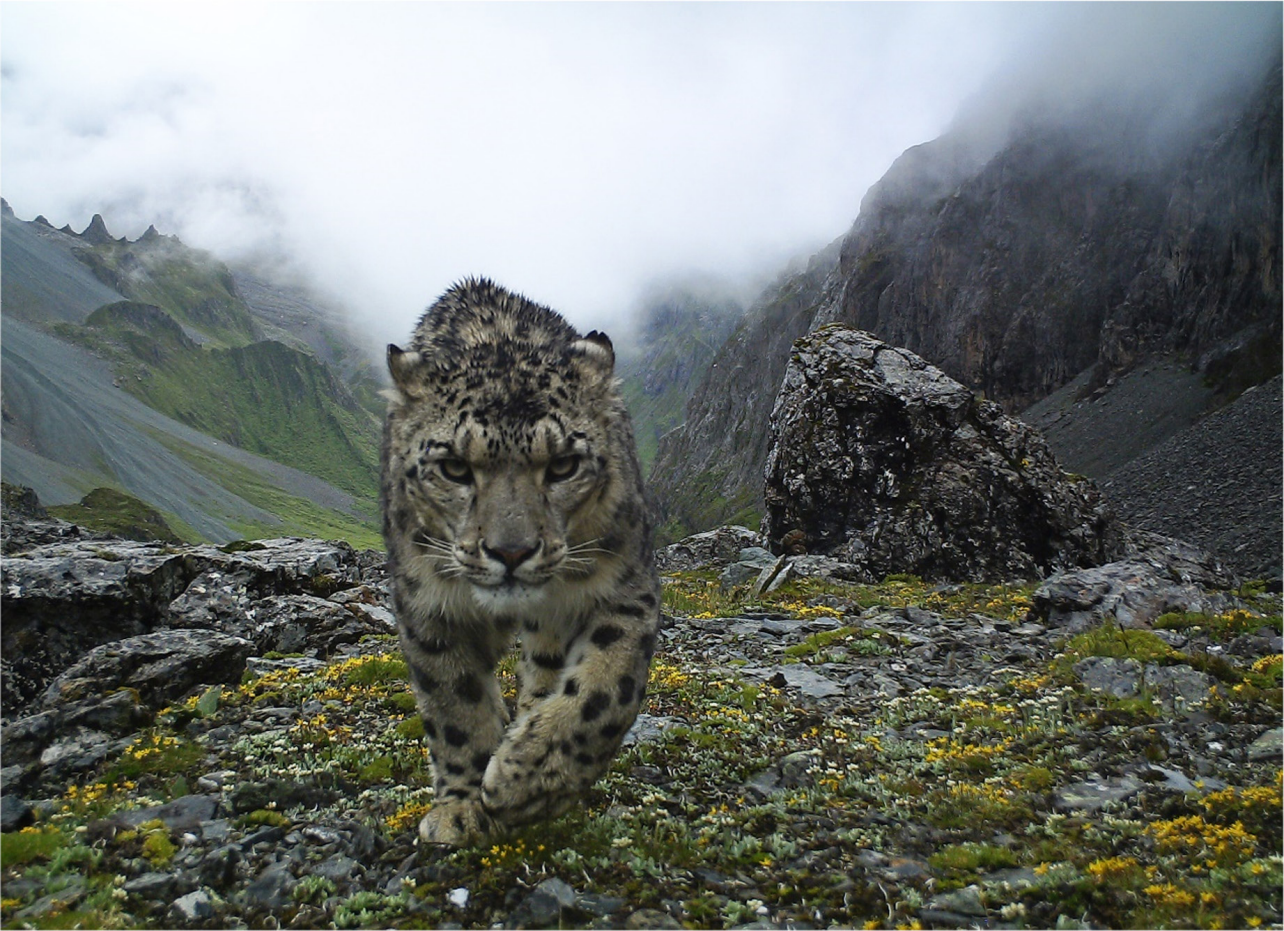
Snow leopard: the top predator in the alpine ecosystem.

Since the earliest snow leopard sighting in 2009, WNNR has continuously monitored its population dynamics. Liu et al. (2019) reported that the highest wild population density of snow leopards in China was found within the reserve, with surveys identifying at least 26 individuals (Figure 1). We also recorded up to three kittens accompanying their mothers simultaneously (as we show in Figure 6), indicating a healthy breeding population of snow leopard.

The Administrative Bureau of WNNR manages the reserve, which includes Wolong Township and Gengda Township. Livestock husbandry serves as a crucial economic income for the local communities, with 4403 agro‐pastoralists relying on livestock grazing for their livelihoods. In 2019, livestock numbers reached 14,971, including 4270 yaks (Liu, Gou, et al., 2021, Liu, Qi, et al., 2021). Yaks roam freely in the mountains for most days of the year and would be sold off in winter to outside markets (Zhang et al., 2017). Considering that herders depend on livestock for their livelihood, it is unlikely that grazing will be completely banned in the reserve in a relatively short period. Recklessly carrying out such policy could create issues affecting people's well‐being and local economics. On balance, the local government should gradually limit the grazing area and intensity to reduce livestock disturbance toward snow leopards and alpine ecosystems. Understanding the impact of grazing on snow leopard and its wild preys in protected areas is the basis for scientific guidance on grazing management and conservation.

### 2.2 Camera traps

**FIGURE 2:**
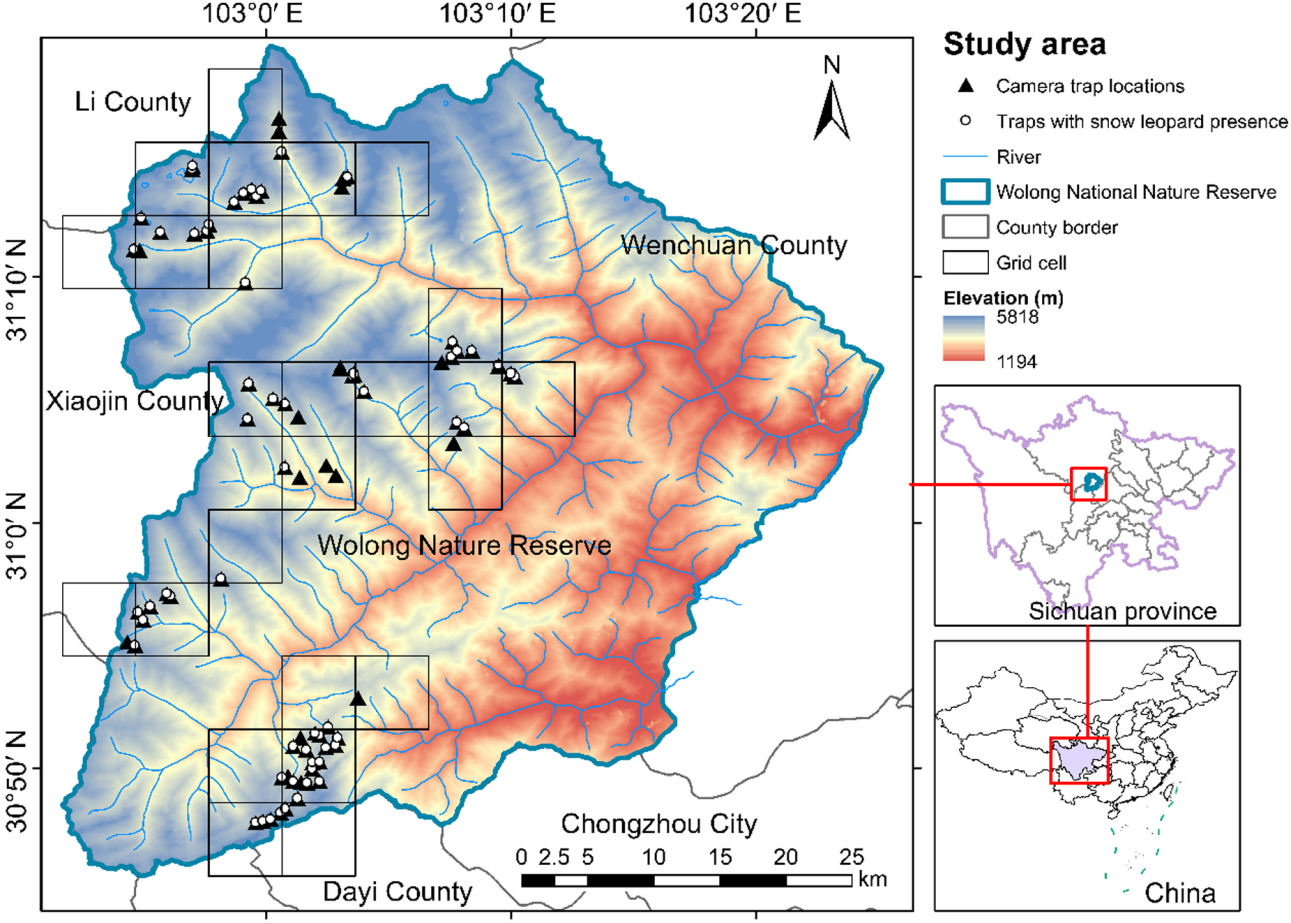
Map of the study area showing camera trap locations and the distribution of snow leopard.

We conducted camera‐trapping surveys from September 2019 to November 2020 using a total of 164 camera traps (Model L710F; Shenzhen Ltl Acorn Electronics Co., Ltd). The study area was divided into 25 grid cells (5 × 5 km, Figure 2), with each camera trap systematically placed within these cells. We determined camera trap locations based on the density of snow leopard tracks and the presence of natural pathways likely to be utilized by wildlife (Nyhus et al., 2016). To avoid spatial autocorrelation leading to inaccurate results, we retained data from only one camera trap in each 1 × 1 km grid, prioritizing those that detected a snow leopard (Alexander et al., 2016). Finally, we used data from 78 camera traps for statistical analyses. The camera‐trapping survey yielded a total effort of 20,861 camera‐days.

### 2.3 Statistics analysis

#### 2.3.1 Detection frequency of animals

Continuous photos of the same species, captured within a 30‐min interval at the same camera site, were considered as an independent detection (Liu et al., 2022). Photos featuring snow leopards, their sympatric large carnivores, potentially important prey, and livestock were retained. Due to the limited number of effective detections for other wildlife species (Table S1), only three species, namely, snow leopard, blue sheep, and yak, were included in the subsequent analysis.

The frequency of independent detections, which indicates the likelihood of sites being visited by a specific species, can be used as a measure of the intensity of habitat use (Feng et al., 2021). We employed the relative abundance index (RAI, average number of independent detections per camera day) to assess the spatial overlap among target species at each site (Yang et al., 2021). The RAI was calculated using the following formula in R 4.0.5:

$${\mathrm{RAI}}_{i,j}=N_{i,j}/D_{j,}$$

where *RAI*_*i, j*_ indicates the relative abundance index of specie *i* at trap *j*; *N*_*i, j*_ indicates the effective detection number of specie *i* at trap *j*; and *D*_*i*_ indicates the effective working days of the camera at trap *j*.

#### 2.3.2 Mann–Whitney *U* test

To assess the utilization interactions among snow leopards, blue sheep, and yak, we conducted a pairwise comparison of each species’ elevation distributions using the Mann–Whitney *U* test (Ahmad et al., 2019). We then stratified the study area into three elevation bands: below 3800 m (forest), 3800–4400 m (alpine scrub meadow), and above 4400 m (bare rock). We compared the detection frequencies of each species across each elevation band. The Mann–Whitney *U* tests were performed using IBM SPSS (version 25.0, IBM Corp). Data visualization was performed using the package “ggplot2” (Wickham, 2016) in R 4.0.5.

#### 2.3.3 Daily activity pattern

To account for seasonal variations in sunrise and sunset times during the survey (Nouvellet et al., 2011), we converted each target species' detection time from clock‐recorded times to relative solar times using the “overlap” package (Ridout & Linkie, 2009) in R 4.0.5. We employed kernel density estimates to analyze the daily activity patterns of each target species based on the time information of each independent record (Ridout & Linkie, 2009). Coefficients of temporal niche overlap, ranging from 0 (no overlap) to 1 (complete overlap), were used to evaluate the temporal overlap between each target species pair in each season. We calculated 95% confidence intervals using a bootstrap method with 1000 iterations (Ridout & Linkie, 2009). We investigated whether significant differences exist in the activity patterns of wild species in areas with and without grazing by utilizing the Watson's 2‐sample test in the “circular (version 0.4‐94)” package (Agostinelli & Lund, 2022) in R 4.0.5.

#### 2.3.4 The structural equation modeling

Structural equation modeling (SEM) was used to evaluate the influence of biological and environmental factors on snow leopard spatial utilization and the influence of yak on snow leopard activities (Rosseel, 2012). Drawing from previous research on habitat selection for snow leopards, blue sheep, and yaks (Ali et al., 2021; Aryal et al., 2016; Chi et al., 2019; Liu et al., 2014; Rashid et al., 2021; Yang et al., 2021), we selected 18 environmental variables, encompassing topographic factors, bioclimatic factors, the enhanced vegetation index (EVI), distance to roads, and vegetation type.

The variance inflation factor (VIF) and Pearson correlation test were then used to check potential collinearity among all environmental variables (Zuur et al., 2009). We retained covariates with VIF < 2 and Pearson's correlation index |*r*| < 0.5 (Zuur et al., 2009). VIF values were calculated in R 4.0.5 using the “see (version 0.7.1)” package (Lüdecke, Ben‐Shachar, et al., 2021, Lüdecke, Patil, et al., 2021), and “performance (version 0.9.1)” (Lüdecke, Ben‐Shachar, et al., 2021, Lüdecke, Patil, et al., 2021).

After the screening, we adopted a total of eight bioenvironmental variables to fit the SEM, including blue sheep and yak detection rates, aspect, slope, land cover type, distance to the road (Dist), EVI, and precipitation coefficient of variation (BIO15).

The bootstrapping method (the naïve bootstrap and the Bollen‐Stine bootstrap) was used to estimate the path coefficients when the SEM model was fitting well (Bollen & Stine, 1992; Rosseel, 2012). Finally, we chose eight indicators to test the fitness of the model: the goodness of fit index (GFI), Bollen's normed‐fit index (NFI), Bollen's comparative fit index (CFI), Bollen's incremental fit index (IFI), the Tucker‐Lewis index (TLI), standardized root‐mean‐square residual (SRMR), root‐mean‐square error of approximation (RMSEA), and the chi‐square test. The model fit was considered good when GFI, NFI, CFI, IFI, and TLI values exceeded 0.95, RMSEA and SRMR values were below 0.05, and the chi‐square test was not significant (*p*‐value > .05). We conducted the SEM analysis in R 4.0.5 using the “lavaan (version 0.6.3)” package (Rosseel, 2012).

## 3 RESULTS

### 3.1 Daily activity pattern

Of 20,861 camera trap days, we obtained a total of 7334 independent mammal photographs, including 705 independent snow leopards at 62 sites. Blue sheep, the predominant prey of snow leopards, were widely distributed throughout the study area, with 2551 independent captures at 74 sites. Yaks were present at 23 sites, with 787 independent captures recorded (Table 1).

**TABLE 1:** Numbers of camera traps that detected targeted animals, independent captures, and detection frequencies (mean ± standard error) of snow leopards, blue sheep, and yaks.

	Snow leopard	Blue sheep	Yaks
Camera traps detected targeted animals	62	74	23
Independent captures (#)	705	2551	787
Detection frequency (#/day)	0.032 ± 0.0042	0.128 ± 0.0174	0.035 ± 0.0132

**FIGURE 3:**
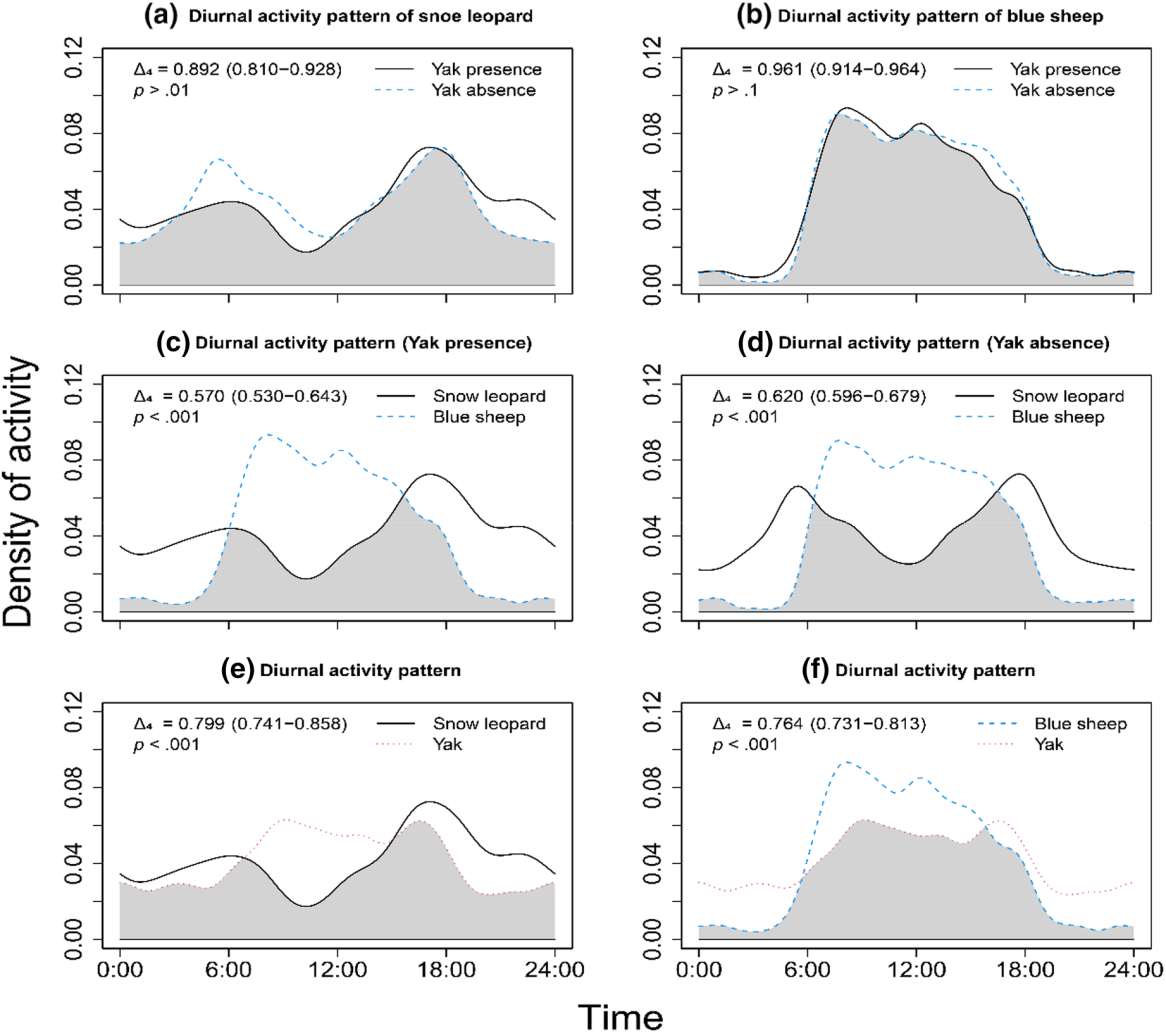
Effect of grazing on the daily activity patterns and the overlap indexes (Δ_4_) of snow leopards and blue sheep. Changes in the activity rhythm of snow leopards and blue sheep under the influence of yaks (a, b). Yaks altered the change in overlap coefficient between snow leopards and blue sheep (c, d). The daily activity patterns and the overlap indexes between the two wild species and yaks (e, f). Numbers in parentheses represent 95% confidence intervals for the overlap indexes. *p*‐values represent the results of the Watson's two‐sample test.

Kernel density function‐based overlap analysis of daily activity rhythms revealed the impact of grazing on the activity patterns of snow leopards and their wild prey (Figure 3). Yak activities were recorded both day and night, with increased activity during the daylight hours, peaking around 09:00 and 17:00. Snow leopards displayed crepuscular activity, peaking around 06:00 and 17:00. Blue sheep were primarily active during the day, with peak activity at morning (around 08:00).

The Watson's test indicated significant differences in the daily activity patterns of snow leopards in the presence or absence of yaks (Δ_4_ = 0.892, *p* > .01, Figure 3a). In areas with yaks, snow leopard activity decreased during the day and increased at dusk. However, grazing did not alter the blue sheep activity pattern (Δ_4_ = 0.961, *p* > .1, Figure 3b), resulting in a reduced temporal overlap with snow leopards from 0.620 (95% CI, 0.596–0.679) to 0.570 (95% CI, 0.530–0.643, Figure 3c,d).

The number of independent detections of wild species used to calculate the overlap index was 1017 and 2239 at sites with or without yaks, respectively.

### 3.2 The interspecific relationships of space use

#### 3.2.1 The relationships in elevational utilization

**FIGURE 4:**
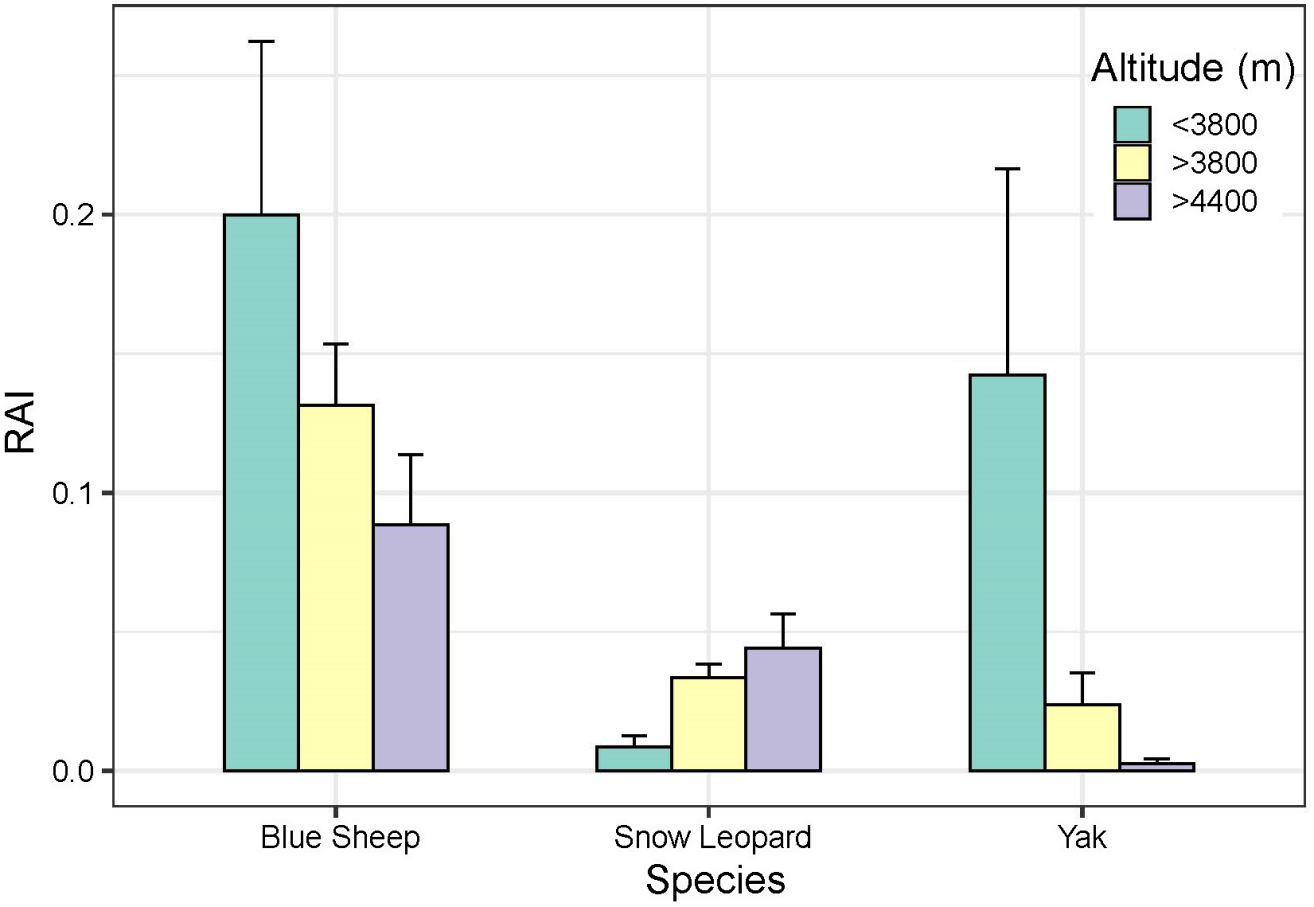
Relative abundance indices (RAI) of the three species at each elevational band. Data are mean + standard error.

No significant differences were observed in the elevation distribution among the three target species (Figure S1). The elevational range of snow leopards remained consistent with that of blue sheep (Figure S1); however, snow leopards exhibited higher detection rates in alpine scree areas compared with other elevations (Figure 4). Both blue sheep and yak demonstrated a preference for lower elevational areas with higher vegetation cover, and yaks exhibited notably low detection rates in alpine screes (Figure 4).

#### 3.2.2 Effects of grazing on habitat selection of snow leopards and blue sheep

**FIGURE 5:**
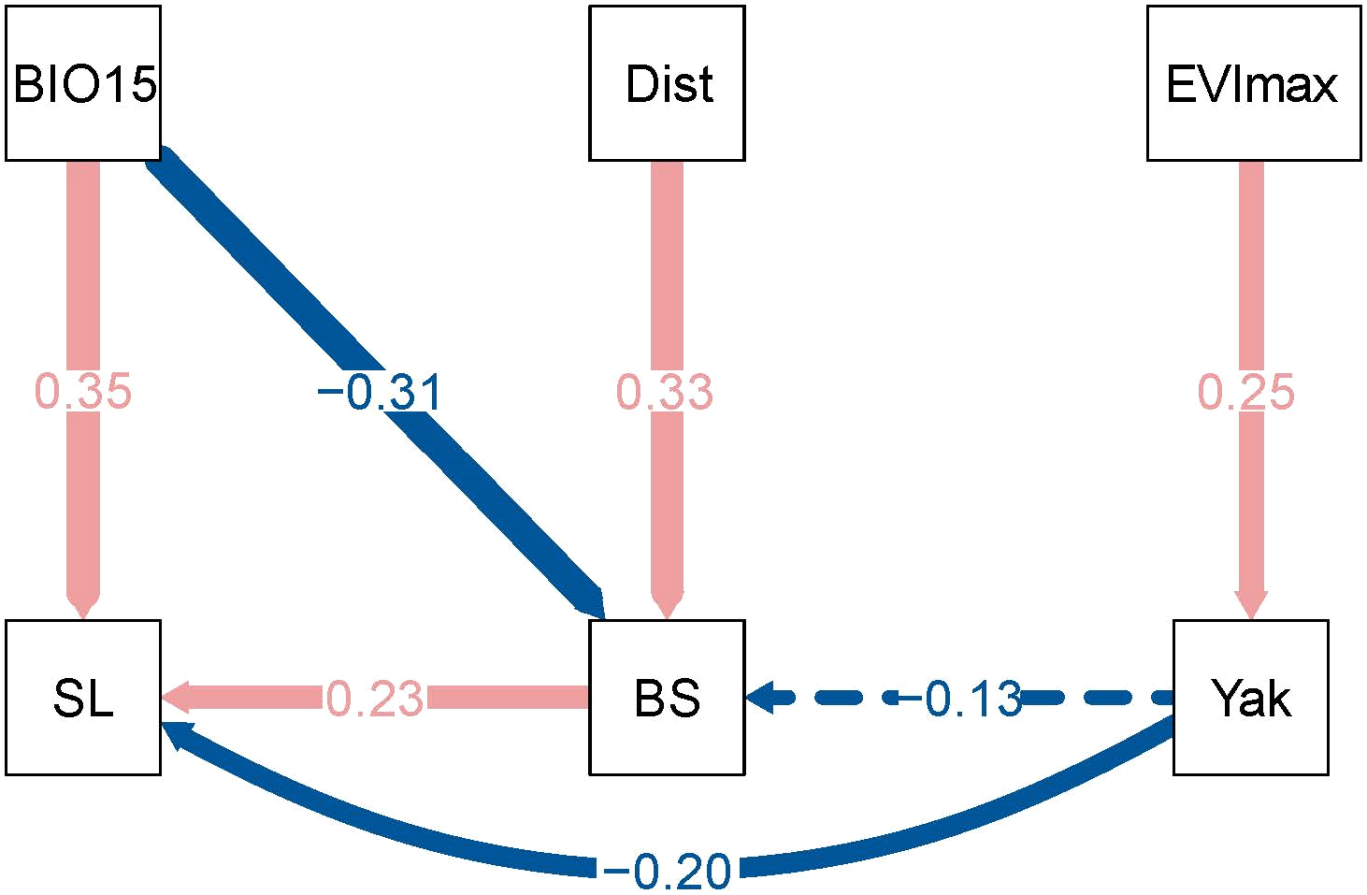
The results of structural equation modeling (SEM) analysis showing the impacts of distance to the road, precipitation coefficient of variation, enhanced vegetation index, terrain ruggedness, detection frequency of yak, and detection frequency of blue sheep (BS) on the detection frequency of snow leopard (SL). The pink line indicates a positive influence and the blue line indicates a negative influence. Significant effects are indicated by solid lines and nonsignificant effects by dashed lines. Arrow widths are proportional to path coefficients. Numbers alongside the paths indicate standardized path coefficients (Coe) and its *p*‐value. Statistics for the model fitness are: GFI = 0.99, NFI = 0.98, CFI = 1.00; IFI = 1.10, TLI = 1.28, *χ* ^2^ = 0.95, *df* = 5, *p* = .97; srmr = 0.02, rmsea = 0.00.

Our SEM results revealed a significant positive correlation between the detection frequencies of snow leopards and blue sheep, while a significant negative correlation between yak and snow leopard detections (Figure 5). Although a negative correlation emerged between the presence of yak and blue sheep was observed, it was not statistically significant (Figure 5).

Regarding environmental factors, the precipitation coefficient of variation exhibited a significant positive effect on the detection rate of snow leopards, yet it negatively affected blue sheep (Figure 5). Furthermore, we found that EVI and distance to the nearest road exerted significant positive influences on the detection frequencies of both yaks and blue sheep (Figure 5).

## 4 DISCUSSION

### 4.1 Survival strategies of snow leopards and blue sheep in predator–prey relationships

Predator–prey interactions are intricate behavioral games of stealth and fear in which responsive prey strive to evade capture by an equally responsive predator (Brown et al., 1999). In the light of optimal foraging theory, both predators and prey seek a balance between foraging costs and opportunities, adopting the foraging strategy that is most conducive to survival and reproduction (Brown et al., 1999). In our study, snow leopards and blue sheep developed distinct survival strategies within this ecological game. Snow leopards were more observed in areas with a higher detection rate of blue sheep (Figure 5), while blue sheep adjusted their activity patterns to minimize predation risk (Figure 3).

Predators tend to concentrate their activities in areas with high predation success but low prey density (Sih, 2005). The hunting success of snow leopard and other solitary, stalking/ambushing predators depends on access to cover to reduce attack distances (Yachi, 2000). Accordingly, snow leopards prefer alpine screes (Figure 4; Qiao et al., 2017; Tang et al., 2017); where their distinctive coloration and pattern provide effective camouflage (Ma et al., 2013). Most importantly, the limited distribution of large carnivores in high‐elevation areas reduces resource competition (Hong et al., 2021). Blue sheep, as the primary food source of snow leopards in the WNNR (Hong et al., 2021), influence snow leopards' movement within the same elevation range (Figure S1). SEM results support the idea that snow leopard activity patterns stem from their preference for blue sheep as wild prey (Figure 5), which align with the behavioral response race theory (Sih, 1984). Therefore, our findings echo prior research that the presence of blue sheep is a critical biological factor determining snow leopard spatial utilization (Filla, Lama, Filla, et al., 2022, Filla, Lama, Ghale, et al., 2022; Suryawanshi et al., 2021). Prey species employ various strategies to mitigate predation risk in numerous ways, such as reducing foraging time, selecting less risky areas or times for foraging, or enhancing vigilance in high‐risk zones (Chitwood et al., 2022; Makin et al., 2017). Blue sheep, despite being present in alpine screes, were more attracted to productive alpine scrub and meadows (Figure 4; Aryal et al., 2013, 2014), which somewhat reduced their probability of encountering snow leopards. Additionally, to survive the pursuit of snow leopards, blue sheep adapted their activities to periods when predators were not active (Figure 3).

In summary, snow leopards and blue sheep choose different activity strategies to cope with the survival pressure of their own. These foraging‐antipredator strategies are manifested by their unique patterns of activity timing and spatial distribution, reflecting the ecological intricacies of interspecific interactions.

### 4.2 Grazing interfered with the habitat use of snow leopards

The results of SEM showed that yak presence significantly influenced snow leopard habitat use. Not surprisingly, we found a significant negative correlation between yak and snow leopard detection rates, indicating a spatial separation between the two species. Grazing commonly has negative impacts on the occupancy of large carnivores (Gervasi et al., 2015; Liu et al., 2022; Soofi et al., 2018), and grazing tends to reduce available habitat types and habitat selectivity for snow leopards (Hong et al., 2021). Moreover, our study revealed a decrease in snow leopard activity during peak yak activity, highlighting the negative effect of free‐ranging livestock on the temporal activity of this large carnivore.

Based on our observations, the negative snow leopard‐yak interaction likely results from the active avoidance by snow leopards. Blue sheep spread throughout the survey area, providing ample wild prey for snow leopards. Consequently, it is reasonable for snow leopards to avoid areas with anthropogenic disturbances like grazing in the circumstance where wild prey availability does not affect habitat suitability. Large carnivores often distance themselves from disturbed areas and reduced diurnal activity to survive in human‐dominated landscapes (Gaynor et al., 2018; Lamb et al., 2020). Although yaks are considered potential prey for snow leopards, most of the free‐ranging yaks in the WNNR are adults with an average weight of 250 kg (Bagchi & Mishra, 2006), which are commonly believed to be too large for snow leopards to kill (Chetri et al., 2017; Hayward & Kerley, 2008; Wang et al., 2018). Theoretically, snow leopards only attack livestock as their secondary prey when wild prey is scarce or hard to find, which is generally during the dry season (winter and spring) when food is limited (Devkota et al., 2013). Even then, they would prefer calves or small livestock (Alexander et al., 2015; Chetri et al., 2017), as the predation on adult yaks was rarely reported (Krofel et al., 2021). Moreover, yaks moving in large groups with dozens of individuals can deter most predators, particularly the solitary predator snow leopard. Another possible explanation for the significant negative correlation between yaks and snow leopards is that human activities associated with grazing (e.g., salt feeding, medication, shearing, etc.) may intimidate snow leopards, similar to how species react to perceived predation risk (Gaynor et al., 2018). These deterrent effects of nonlethal disturbance by humans on wildlife are quite common among large carnivores (Gaynor et al., 2018; Wilkinson et al., 2020). Thus, snow leopards may perceive yaks as a flag for human‐related risk and thus avoid direct encounters with yaks (Wolf & Ale, 2009).

Generally, livestock grazing can influence the trophic structure of ecological communities, in particular affecting trophic levels that directly rely on plants, such as ungulates (Filazzola et al., 2020; Schieltz & Rubenstein, 2016). In terms of site usage fluctuations, wild ungulates tend to reduce pasture utilization or relocate to steeper slopes or less favorable habitat when livestock are present (Schieltz & Rubenstein, 2016). Yang et al. (2021) theorize that spatial suppression of wild prey by livestock is a major constraint on snow leopard population distribution at Gongga Mountain. Temporally, Lahkar et al. (2020) suggest that free‐ranging livestock could lead to diminished diurnal activity levels among ungulates (Lahkar et al., 2020). However, we found no significant evidence for decreased daytime activity of blue sheep in areas with yak presence. It is worth noting that the temporal overlap between snow leopards and blue sheep in grazing zones is reduced, potentially affecting snow leopard predation patterns. Spatially, we found that the presence of yaks had a nonsignificant negative impact on blue sheep habitat utilization (Figure 5), suggesting a degree of spatial coexistence. According to the niche packing theory, high productivity levels allow for finer partitioning of available ecological niches among species, thus enabling the coexistence of species with similar niches in the same geographic region (Klopfer & Macarthur, 1961; Nordberg & Schwarzkopf, 2019). Thus, we speculated that the abundant alpine shrubs and meadow vegetation (Wei et al., 2019) would be sufficient to support habitat sharing between blue sheep and yaks, implying that food availability may not be a limiting factor for their coexistence.

### 4.3 Conservation management implications

**FIGURE 6:**
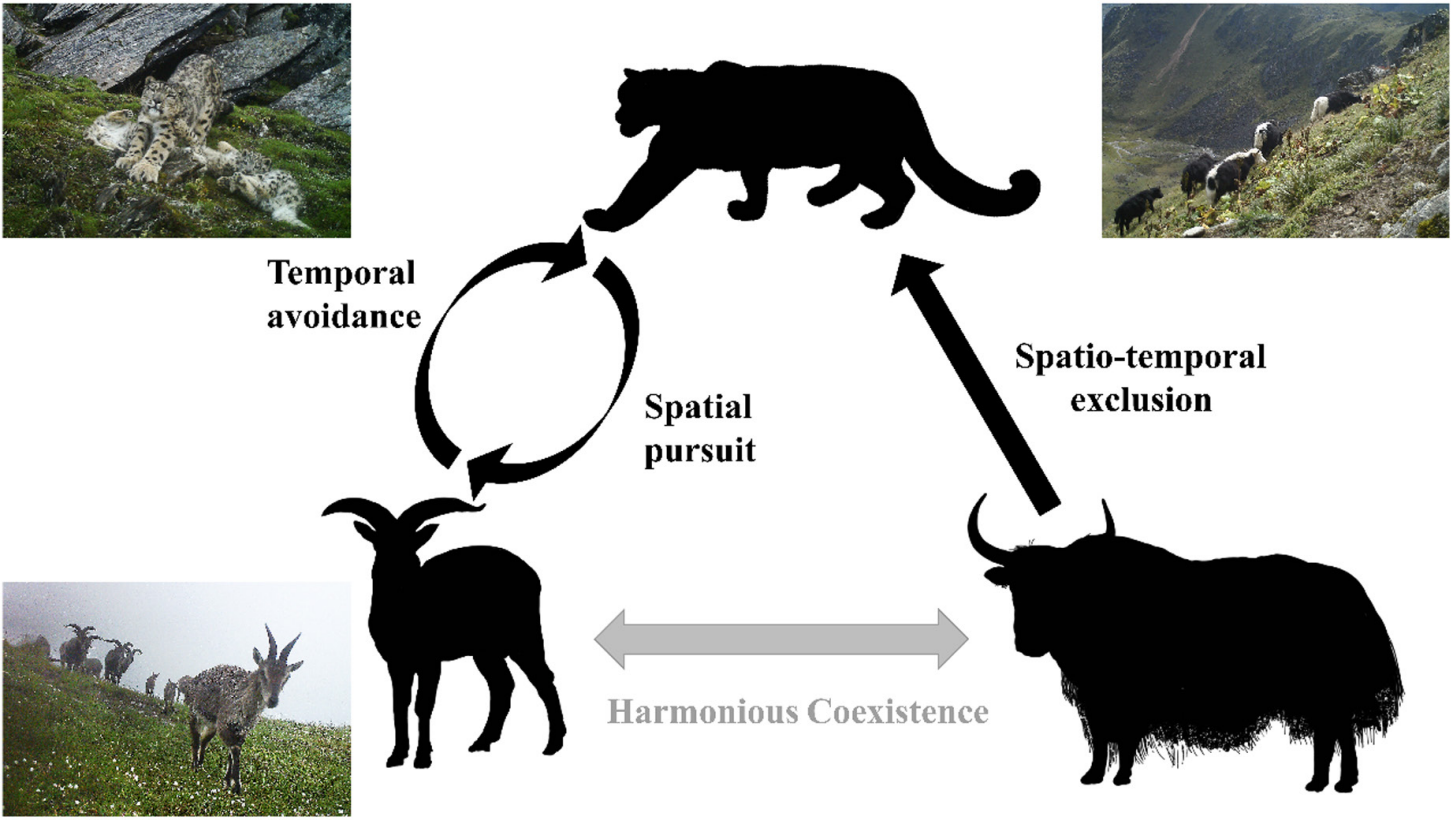
Schematic representation of the interactions among snow leopards, blue sheep, and yaks within the WNNR and photographs of the species taken in the field.

Our study revealed that snow leopards exhibit multilevel avoidance behaviors in response to the intrusion of free‐ranging livestock, and we discussed potential explanations for these effects (Figure 6). We also observed a high degree of similarity in ecological niches between yaks and blue sheep (Figure 6). However, due to the abundance of local resources, intense competitive exclusion between these species has not yet occurred. Given the high dependence of snow leopards on large wild prey (Suryawanshi et al., 2017), it is crucial to ensure an adequate population of wild ungulates. We emphasize the importance of monitoring wild ungulate population dynamics under the influences of free‐ranging yaks.

Numerous studies have focused on ecological networks in forest ecosystems, such as tigers inhabiting the forests of South Asia (Penjor et al., 2019; Thatte et al., 2021). By contrast, our research investigated snow leopards, which inhabit high‐altitude regions characterized by lower vegetation coverage and more fragile environments (Hong et al., 2021). Specifically, our study area is a typical alpine canyon region with lower connectivity compared with snow leopard habitats in Sanjiangyuan (Yan et al., 2019). The intricate topography of the alpine canyon region results in many small, isolated patches of diverse landscapes. This fragmentation of suitable snow leopard habitats necessitates their traversal through low‐altitude areas with more human activity, exposing them to intense anthropogenic interference. To make matters worse, free‐ranging grazing that allows livestock to roam relatively more extensively in these fragile habitats exacerbates the disturbance. Although blue sheep are undoubtedly a staple food source, local livestock has been added to snow leopards' diet (Hong et al., 2021), contributing to widespread human‐animal conflicts without adequate compensation programs (Xu et al., 2019). With the increasing scale of grazing in the reserve (Liu, Gou, et al., 2021, Liu, Qi, et al., 2021), it is predictable that livestock‐induced pressure on wildlife will be intensified (Pudyatmoko, 2017) and the human‐animal conflict will be exacerbated (Johansson et al., 2015). If so, the impacts of grazing on blue sheep and snow leopards are expected to be contingent upon livestock numbers and pasture management (Maheshwari & Sathyakumar, 2020). We thus contend that controlled grazing in snow leopard habitats is crucial in multiple aspects.

The ideal conservation policy would entail a complete ban on free‐ranging livestock. However, finding alternative livelihoods is challenging since local communities rely heavily on livestock (Liu, Gou, et al., 2021, Liu, Qi, et al., 2021). We propose that local governments should promote the transition to nonagricultural economies, such as migrant employment and ecotourism. Sustainable livelihoods through conservation‐friendly land use, community‐based wildlife management, and co‐management could offer a promising way to replace and reduce the impact of pastoralism, ultimately benefiting wild ungulates and carnivores (Kachel et al., 2017). Encouraging local communities to participate in conservation efforts can also provide employment opportunities and foster the implementation of conservation policies (Peng & Wang, 2017).

In this study, we explored the temporal and spatial interactions among yaks, snow leopards, and blue sheep to assess how snow leopards, an apex predator and indicator of high‐altitude ecosystem health, cope with environmental challenges at the eastern edge of their range. We found that snow leopards spatially followed blue sheep, while blue sheep cleverly used the time when snow leopards were inactive to avoid predation; contrary to expectations, yaks did not pose a significant threat to blue sheep but directly affected the spatiotemporal distribution of snow leopards. Our results provided scientific guidance for coordinating grazing and conservation efforts, as well as for developing targeted management strategies.

## AUTHOR CONTRIBUTIONS

**Jiaxin Li:** Conceptualization (equal); formal analysis (lead); writing – original draft (equal); writing – review and editing (equal). **Xiaogang Shi:** Data curation (equal); investigation (equal); resources (equal). **Xingcheng He:** Conceptualization (equal); writing – original draft (equal); writing – review and editing (equal). **Dongrui Li:** Formal analysis (supporting); writing – review and editing (equal). **Qiang Hu:** Data curation (equal); investigation (equal); resources (equal). **Yanni Zhang:** Formal analysis (supporting). **Jianghong Ran:** Conceptualization (equal); funding acquisition (equal); project administration (lead); writing – review and editing (equal).

## FUNDING INFORMATION

This work was supported by the National Natural Science Foundation of China No. 31970495, the Fundamental Research Funds for the Central Universities No. SCU2022D003, and the Project of dynamic monitoring of snow leopards in Wolong National Nature Reserve.

## CONFLICT OF INTEREST STATEMENT

The authors declare no conflict of interest.

## PERMISSION TO REPRODUCE MATERIALS FROM OTHER SOURCES

None.

## Supporting information


Appendix S1:

## Data Availability

The original contributions presented in this study are included in the article/Supplementary Material; further inquiries can be directed to the corresponding author.
