# Supplementary material for: Free‐ranging livestock affected the spatiotemporal behavior of the endangered snow leopard (Panthera uncia)
Source: Ecol Evol. 2023 Apr 19;13(4):e9992. doi: 10.1002/ece3.9992 (PMC10115902; doi:10.1002/ece3.9992)
Supplement: Supplementary file 1 — Appendix S1: [file ECE3-13-e9992-s001.docx]

Supplementary

Additional data relevant to this paper are listed below.

Table S1 Number of independent detections, number of sites occurred, and relative abundance indexes (RAI) per camera-day of snow leopards and their sympatric mammals according to camera-trapping survey during 2019-2020. In which, RAI is the total number of effective detections/total camera days for each site.

| **Species** | **No. site occurred** | **No. detections** | **RAI** |
| --- | --- | --- | --- |
| Leopard Cat (*Prionailurus bengalensis*) | 7 | 8 | 0.010 |
| Leopard (*Panthera pardus*) | 2 | 2 | 0.005 |
| **Snow Leopard (*Panthera uncia*)** | **61** | **705** | **0.047** |
| Red Fox (*Vulpes vulpes*) | 42 | 229 | 0.026 |
| Wolf (*Canis lupus*) | 2 | 5 | 0.009 |
| Red Panda (*Ailurus fulgens*) | 4 | 4 | 0.007 |
| Asiatic Black Bear (*Ursus thibetanus*) | 8 | 14 | 0.012 |
| Yellow-throated Marten (*Martes flavigula*) | 4 | 6 | 0.015 |
| Beech Marten (*Martes foina*) | 36 | 160 | 0.023 |
| Altai Weasel (*Mustela altaica*) | 18 | 25 | 0.008 |
| Greater Hog Badger (*Arctonyx collaris*) | 44 | 295 | 0.033 |
| Siberian Weasel (*Mustela sibirica*) | 3 | 3 | 0.008 |
| Tufted Deer (*Elaphodus cephalophus*) | 4 | 4 | 0.007 |
| Sambar (*Rusa unicolor*) | 14 | 170 | 0.069 |
| Forest Musk Deer (*Moschus berezovskii*) | 4 | 4 | 0.009 |
| Wild Boar (*Sus scrofa*) | 12 | 33 | 0.017 |
| Takin (*Budorcas taxicolor*) | 25 | 326 | 0.064 |
| **Blue Sheep (*Pseudois nayaur*)** | **74** | **2551** | **0.141** |
| Chinese goral (*Naemorhedus griseus*) | 18 | 51 | 0.020 |
| Chinese serow (*Capricornis milneedwardsii*) | 5 | 6 | 0.013 |
| Golden snub-nosed monkey (*Rhinopithecus roxellana*) | 7 | 23 | 0.022 |
| Himalayan Marmot (*Marmota himalayana*) | 46 | 454 | 0.044 |
| Pika (*Ochotona* spp.) | 18 | 67 | 0.022 |
| **Yak (*Bos grunniens*)** | **23** | **787** | **0.161** |
| Sheep (*Ovis Aries*) | 3 | 69 | 0.177 |
| Horse (*Equus caballus*) | 2 | 84 | 0.268 |
| Human (*Homo sapiens*) | 5 | 11 | 0.009 |


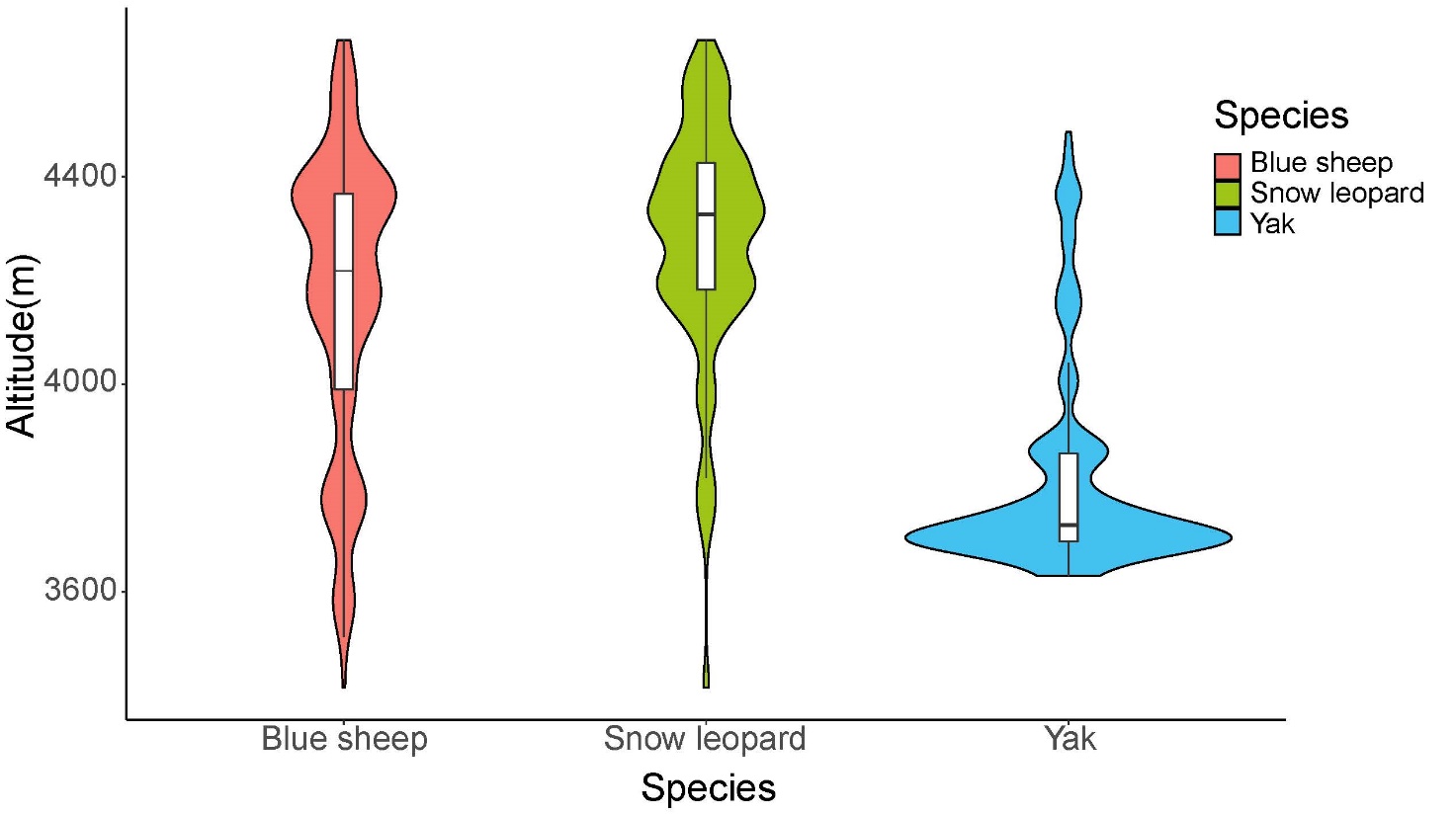


Figure S1 Elevation distribution of snow leopards, blue sheep, and yaks. The width of the violin map shows the effective detection frequency of the corresponding species at each altitude. The Mann-Whitney U test showed that the differences in elevation distribution between yaks and snow leopards (Z = 1.715, *p*-value = 0.086), yaks and blue sheep (Z = 1.095, *p*-value = 0.274), and snow leopards and blue sheep (Z = -0.714, *p*-value = 0.475) were all non-significant.
